# Supplementary material for: Renal Endothelial Single-Cell Transcriptomics Reveals Spatiotemporal Regulation and Divergent Roles of Differential Gene Transcription and Alternative Splicing in Murine Diabetic Nephropathy
Source: Int J Mol Sci. 2024 Apr 13;25(8):4320. doi: 10.3390/ijms25084320 (PMC11050020; doi:10.3390/ijms25084320)
Supplement: Supplementary file 1 [file ijms-25-04320-s001.zip › Supplemental Figures and Tables final.pdf]

Supplemental Figures and Tables for:

Renal endothelial single-cell transcriptomics reveals spatiotemporal regulation and divergent roles of differential gene transcription and alternative splicing in murine diabetic nephropathy

Alex-Xianghua Zhou[1,§], Marie Jeansson[2,3,§], Liqun He[2,3], Leif Wigge[4], Pernilla Tonelius[1], Ramesh Tati[1], Linda Cederblad[1], Lars Muhl[2], Martin Uhrbom[1,2], Jianping Liu[2], Anna Björnson Granqvist[1], Lilach O. Lerman[5], Christer Betsholtz[2,3,#], Pernille B. L. Hansen[1,#]

[1]*Research and Early Development, Cardiovascular, Renal and Metabolism, BioPharmaceuticals R&D, AstraZeneca, Gothenburg, Sweden*
[2]*Department of Medicine Huddinge, Karolinska Institutet, Huddinge, Sweden*
[3]*Department of Immunology, Genetics and Pathology, Uppsala University, Uppsala, Sweden*
[4]*Data Sciences and Quantitative Biology, Discovery Sciences, BioPharmaceuticals R&D, AstraZeneca, Gothenburg, Sweden*
[5]*Division of Nephrology and Hypertension, Mayo Clinic, Rochester, MN, United States*
[§,#] *Equal contribution*

Correspondence: Pernille B. L. Hansen, Cardiovascular, Renal and Metabolism, AstraZeneca AB R&D, Pepparedsleden 1, 431 50 Mölndal, Sweden. Email: pernille.laerkegaardhansen@astrazeneca.com

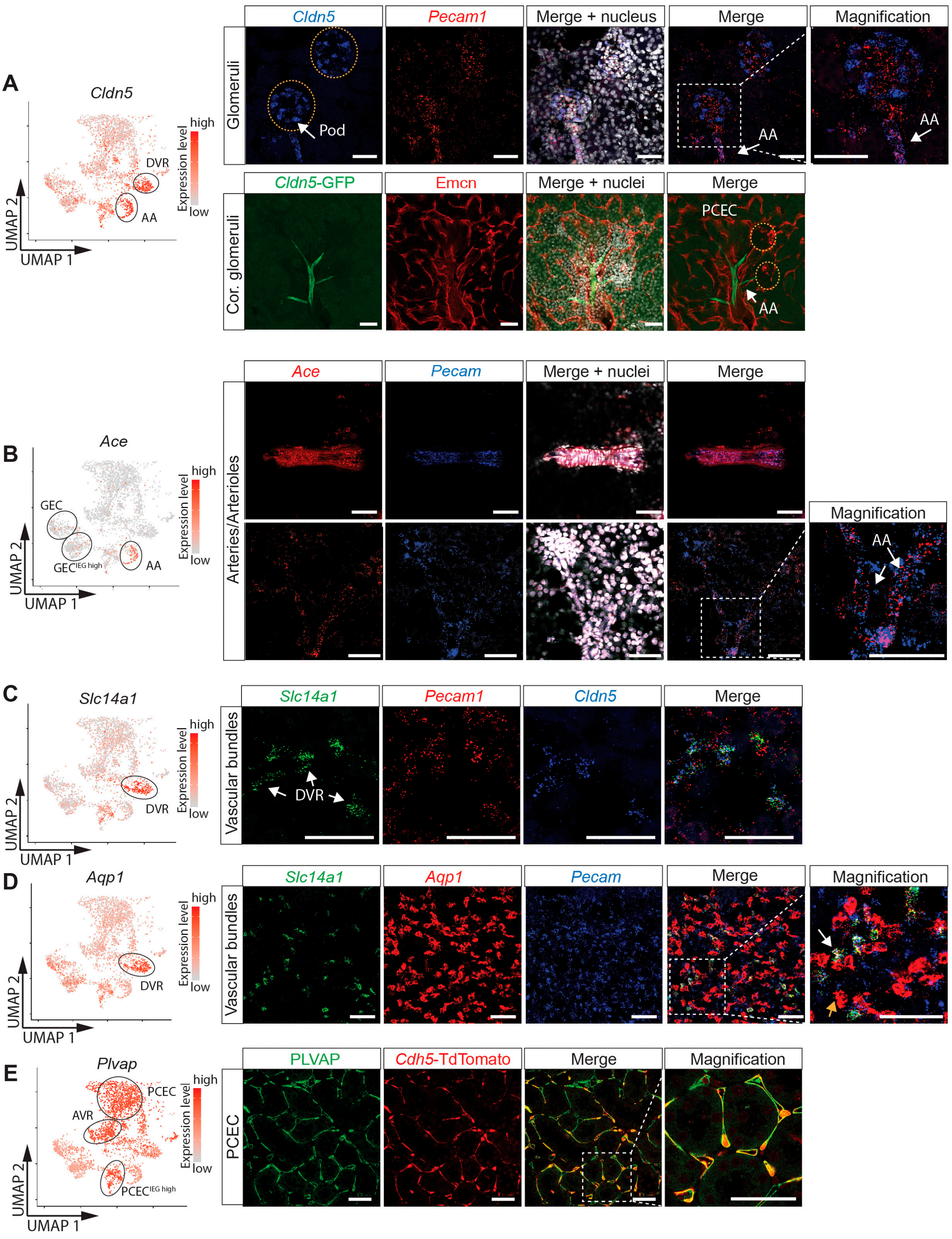

A
Cldn5
DVR
AA
UMAP 1
UMAP 2
Expression level
high
low
Glomeruli
Cldn5
Pecam1
Merge + nucleus
Merge
Magnification
Pod
AA
Cor. glomeruli
Cldn5-GFP
Emcn
Merge + nuclei
PCEC
B
Ace
GEC
GEC IEG high
Arteries/Arterioles
Pecam
C
Slc14a1
Vascular bundles
Pecam1
D
Aqp1
E
Plvap
PCEC
AVR
PCEC IEG high
PLVAP
Cdh5-TdTomato

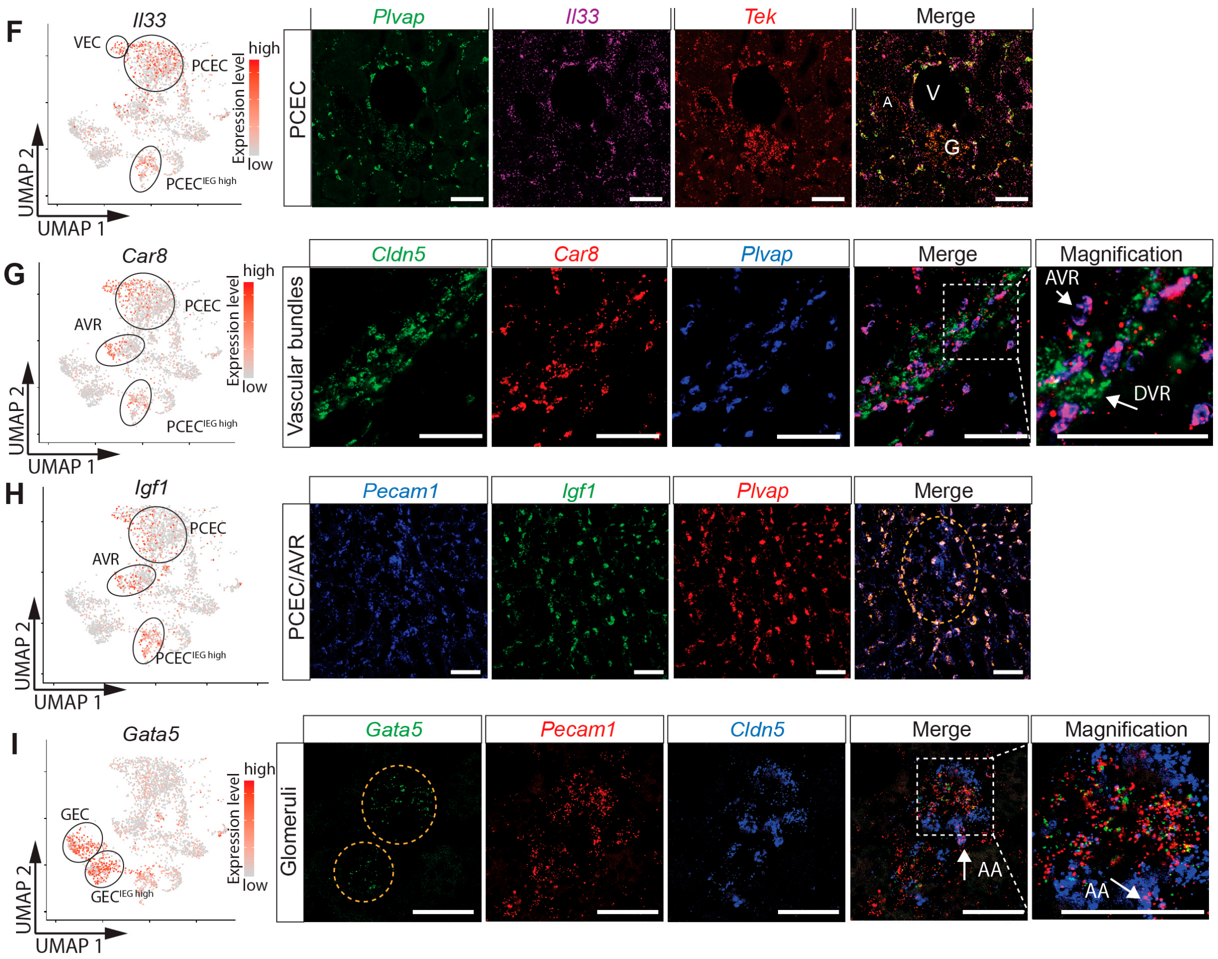

Figure S1. Validation of vascular subpopulations in C57BL6/J mice. (A) UMAP of *Cldn5* and RNA-ISH for *Cldn5* in arteries and afferent arterioles (AA) together with *Pecam1* expression. *Cldn5* expression can also be seen in podocytes (Pod), excluded from *Pecam1*+ cells in the glomerulus, but colocalizing with *Pecam1* in the afferent arteriole (AA). *Cldn5*-GFP reporter mice showed GFP expression in arterioles and afferent arterioles (AA) but not in podocytes. Orange circles indicate glomeruli. (B) UMAP of *Ace* and RNA-ISH identified *Ace* as a novel marker for arteries and afferent arteriole (AA). Lower panel shows afferent arteriole (AA) with *Ace* expression branching to two glomeruli. (C) UMAP of the known descending vasa recta (DVR) marker *Slc14a1*. *Slc14a1* RNA-ISH show colocalization of *Slc14a1* with *Cldn5* in DVR (arrows). (D) UMAP of *Aqp1* and RNA-ISH for *Aqp1* show *Aqp1* expression in DVR (white arrow), but at much lower levels than in epithelial cells (yellow arrow) of the medulla. (E) UMAP of *Plvap* and immunohistochemistry staining of PLVAP show its expression in PCEC together with *Cdh5*-driven TdTomato. (F) UMAP of *Il33* and RNA-ISH for *Il33* show its expression in PCEC, VEC (V, veins) but not in glomeruli (G) or arteries (A). (G) UMAP of *Car8* and RNA-ISH for *Car8* show its expression in *Plvap*+ ascending vasa recta (AVR) but not in *Cldn5*+ DVR in vascular bundles. (H) UMAP of *Igf1* and RNA-ISH for *Igf1* show its expression in *Plvap*+ AVR in vascular bundles indicated by yellow circle and in PCEC. (I) UMAP of *Gata5* and RNA-ISH for *Gata5* show specific expression in GEC, glomeruli marked with orange circle. Scale bar = 50 µm.

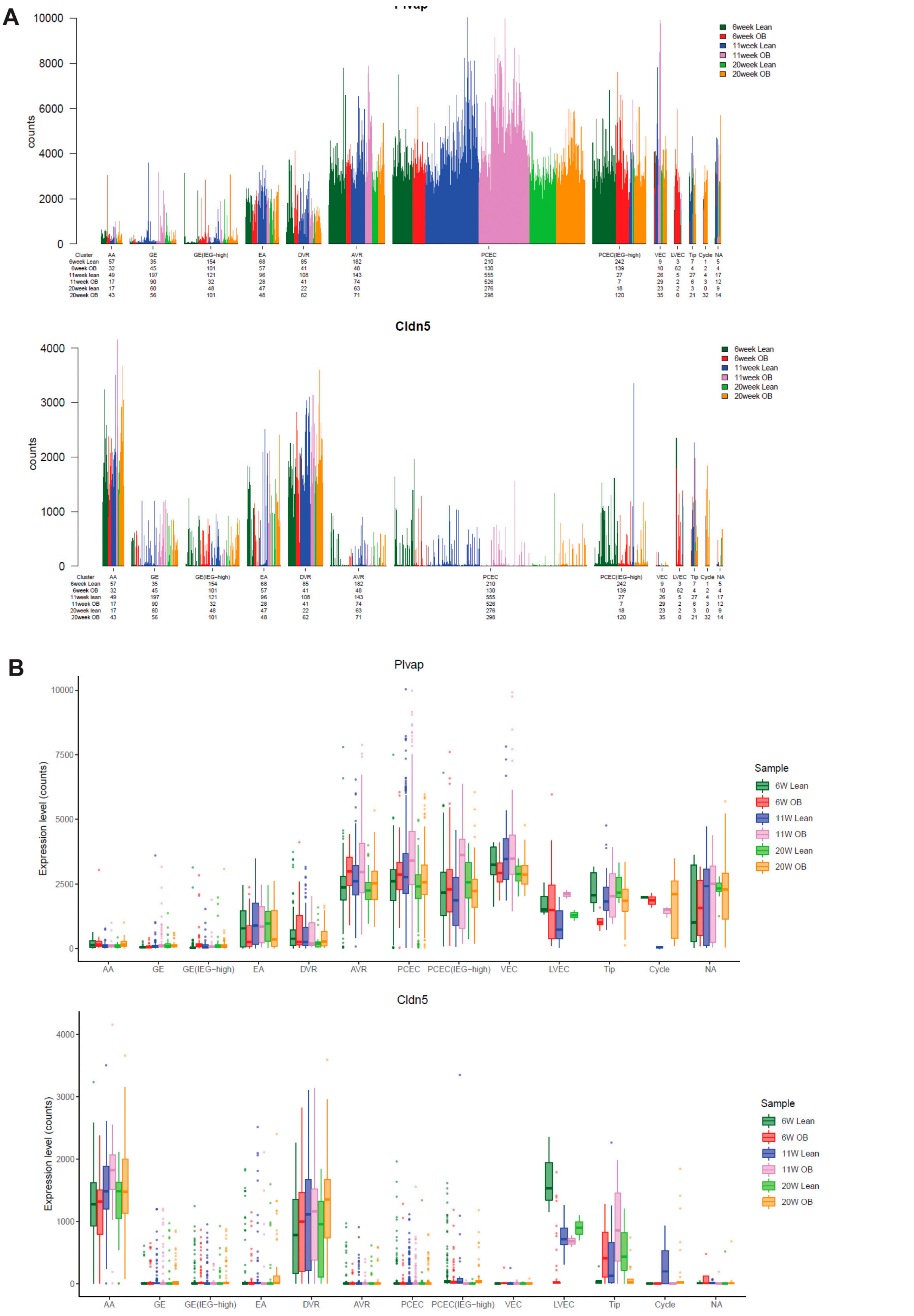

Figure S2. Searchable database. The searchable database show counts for individual cells in a bar plot illustrated by one line (A) or as median (B) for each group and EC population.

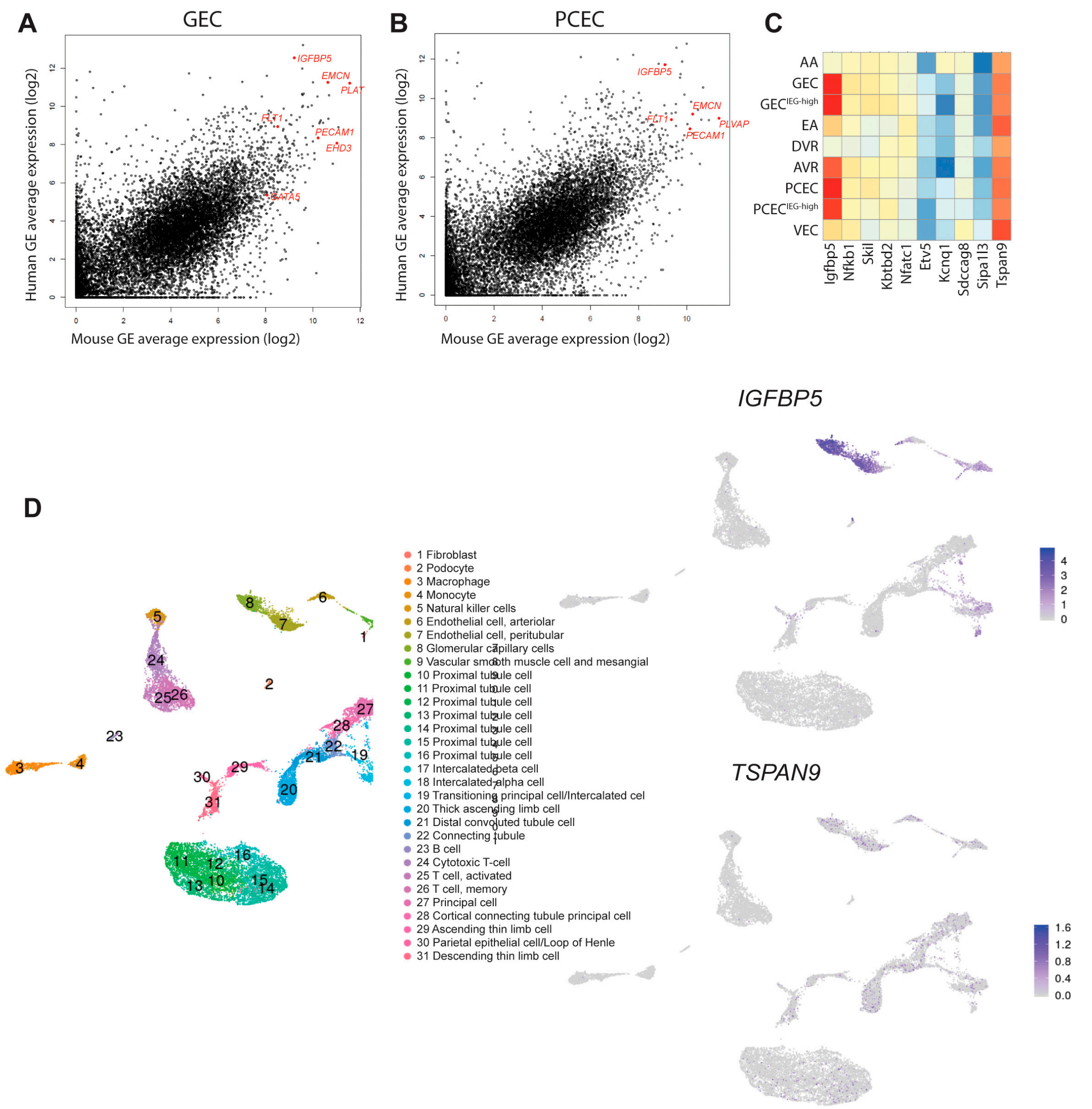

Figure S3. Mouse and human expression correlation. (A, B) Comparison of human GEC (A) and PCEC (B) to the same EC population in the current dataset. (C) 10 of 53 genes associated with human DKD by GWAS could be mapped to EC expression in the current dataset. (D) *IGFBP5* and *TSPAN9* in human EC show similar expression pattern as in mouse.

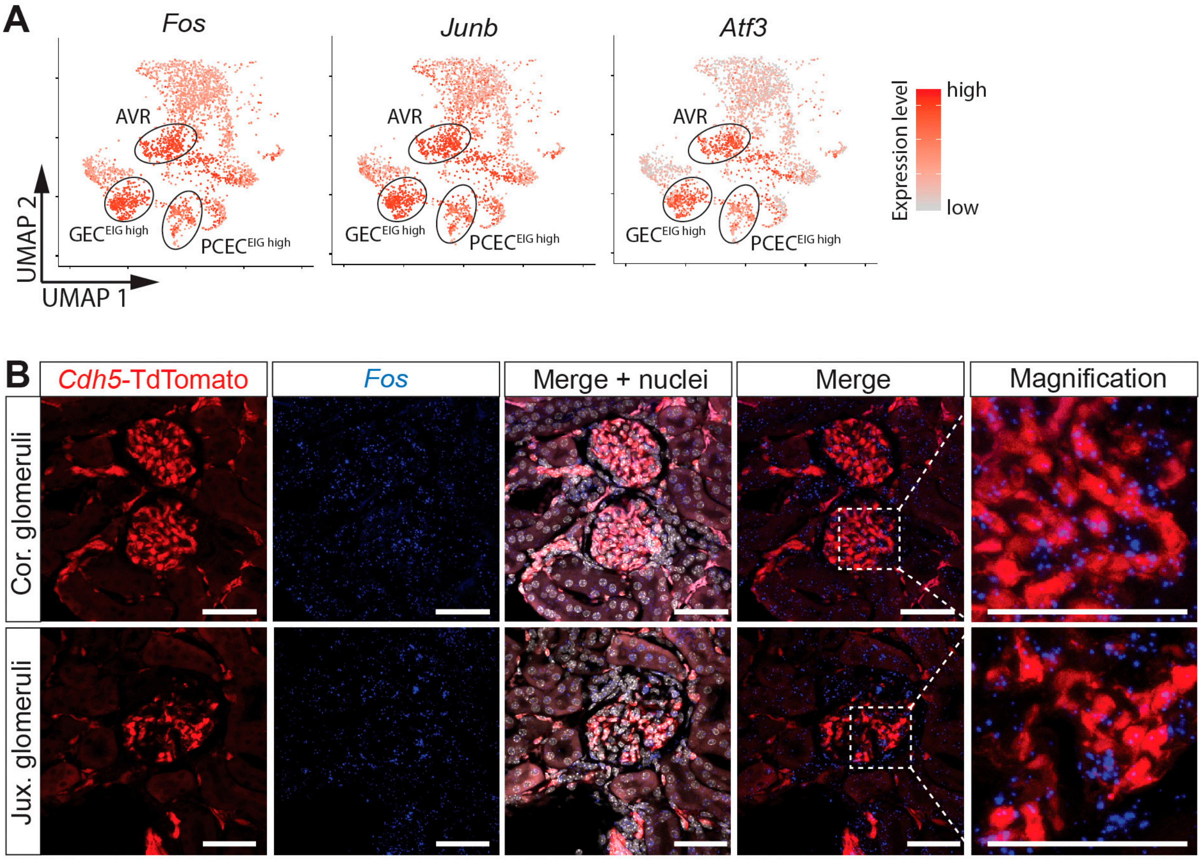

Figure S4. Immediate early gene (IEG) expression. (A) UMAP's for the IEG's *Fos*, *Junb*, and *Atf3* in BTBR*ob/ob* and Lean mice. (B) RNA-ISH for *Fos* in cortical and juxtamedullary glomeruli showing endothelial cells with *Cdh5*-TdTomato (red) in C57BL6/J mice. Scale bars = 50 µm.

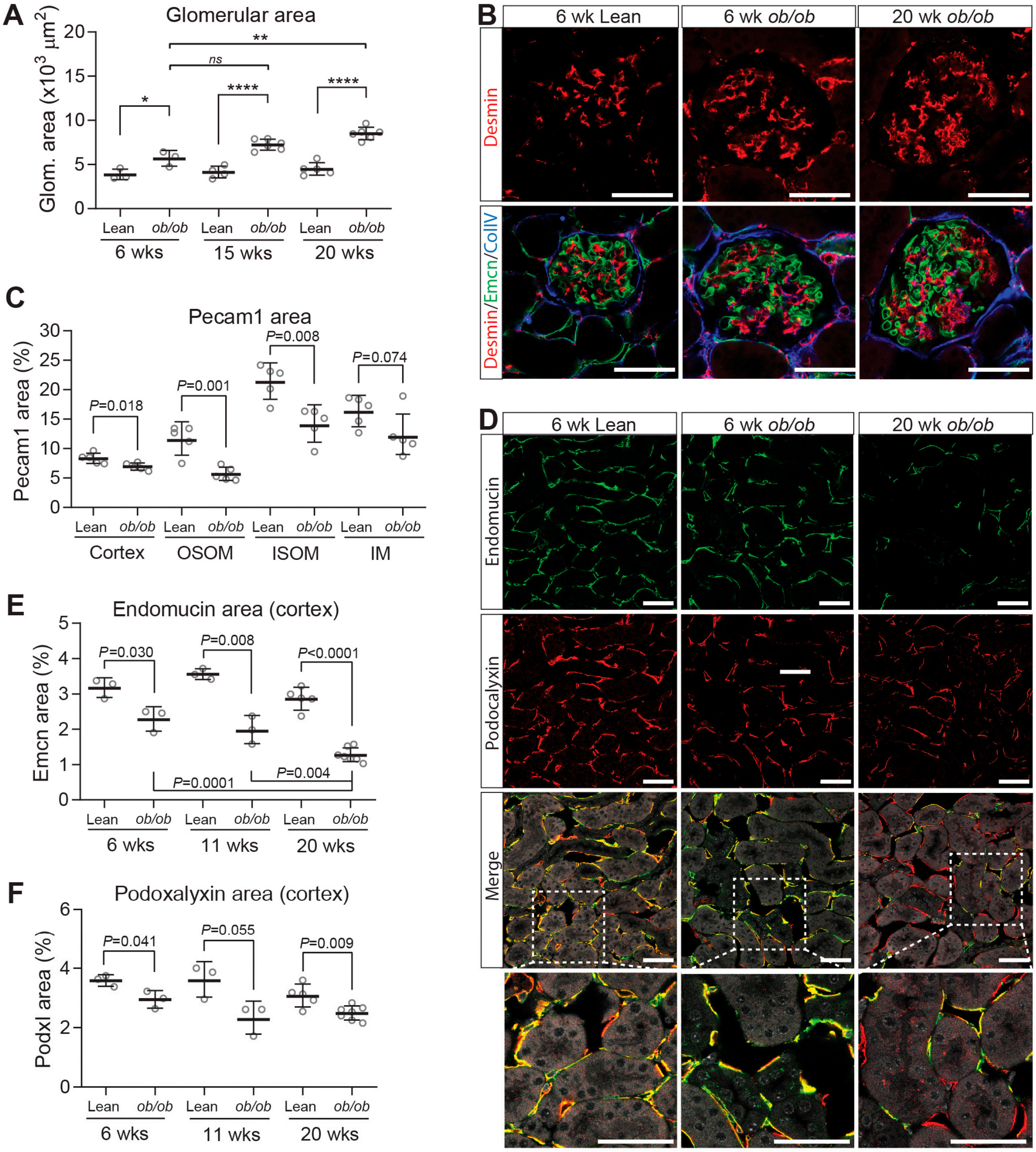

Figure S5. Diabetic kidney disease in BTBR*ob/ob* mice. (A, B) Quantification of glomerular area from immunohistochemistry of glomeruli for desmin, collagen IV, and endomucin at indicated timepoints in non-diabetic (Lean) and diabetic (*ob/ob*) mice. (C) Quantification of PECAM1 stained area in different regions of the kidney; cortex, outer stripe of medulla (OSOM), inner stripe of medulla (ISOM), and inner medulla (IM) in 11-week-old non-diabetic (Lean) and diabetic (*ob/ob*) mice. (D) Representative images and (E, F) quantification of capillary density from immunohistochemistry for endomucin and podocalyxin in renal cortex of non-diabetic (Lean) and diabetic (*ob/ob*) mice at indicated ages. Data expressed as mean ± SD. Scale bar = 50 μm. *p<0.05, **p<0.01, and ****p<0.0001.

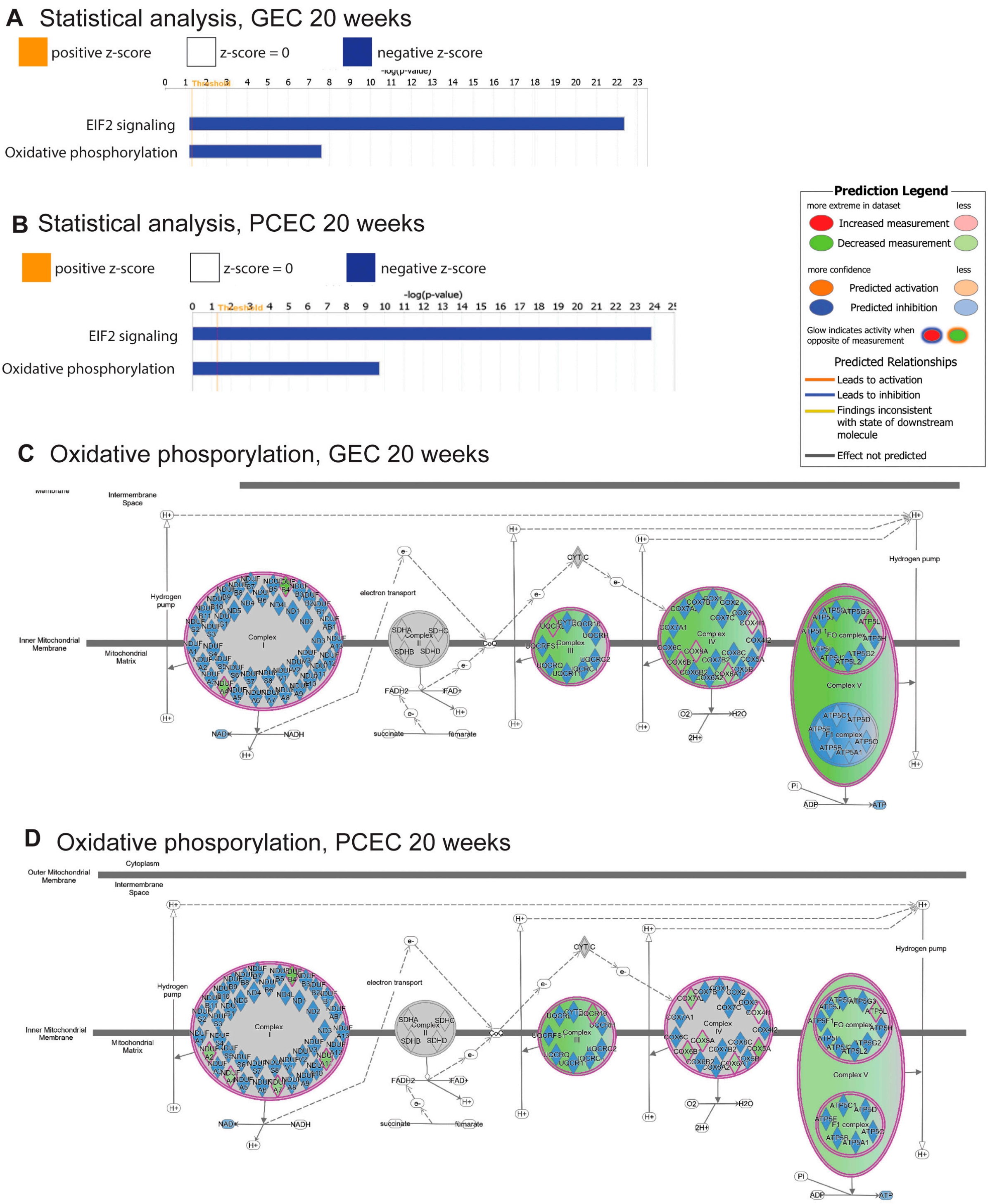

Figure S6. Statistics of the top enriched pathways and oxidative phosphorylation in BTBR*ob/ob* GEC and PCEC at 20 weeks. Statistical analysis of EIF2 signaling and oxidative phosphorylation in GEC (A) and PCEC (B) at 20 weeks based on DEGs comparing non-diabetic (Lean) and diabetic (*ob/ob*) mice. Schematic diagram of oxidative phosphorylation in GEC (C) and PCEC (D) at 20 weeks.

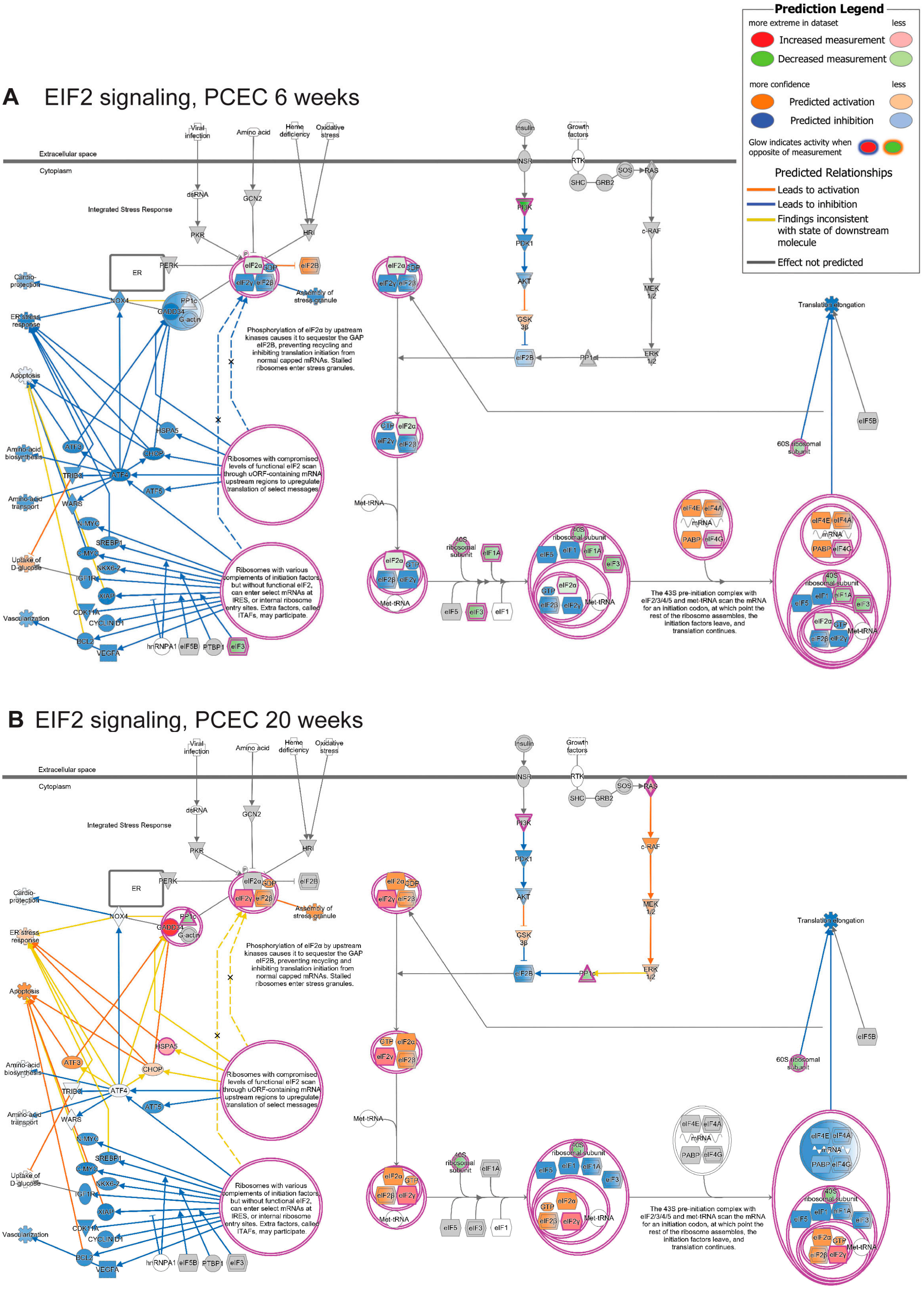

Figure S7. EIF2 signaling in BTBR*ob/ob* PCEC at 6 and 20 weeks. Schematic diagram of EIF2 signaling in PCEC at 6 weeks (A) and 20 weeks (B).

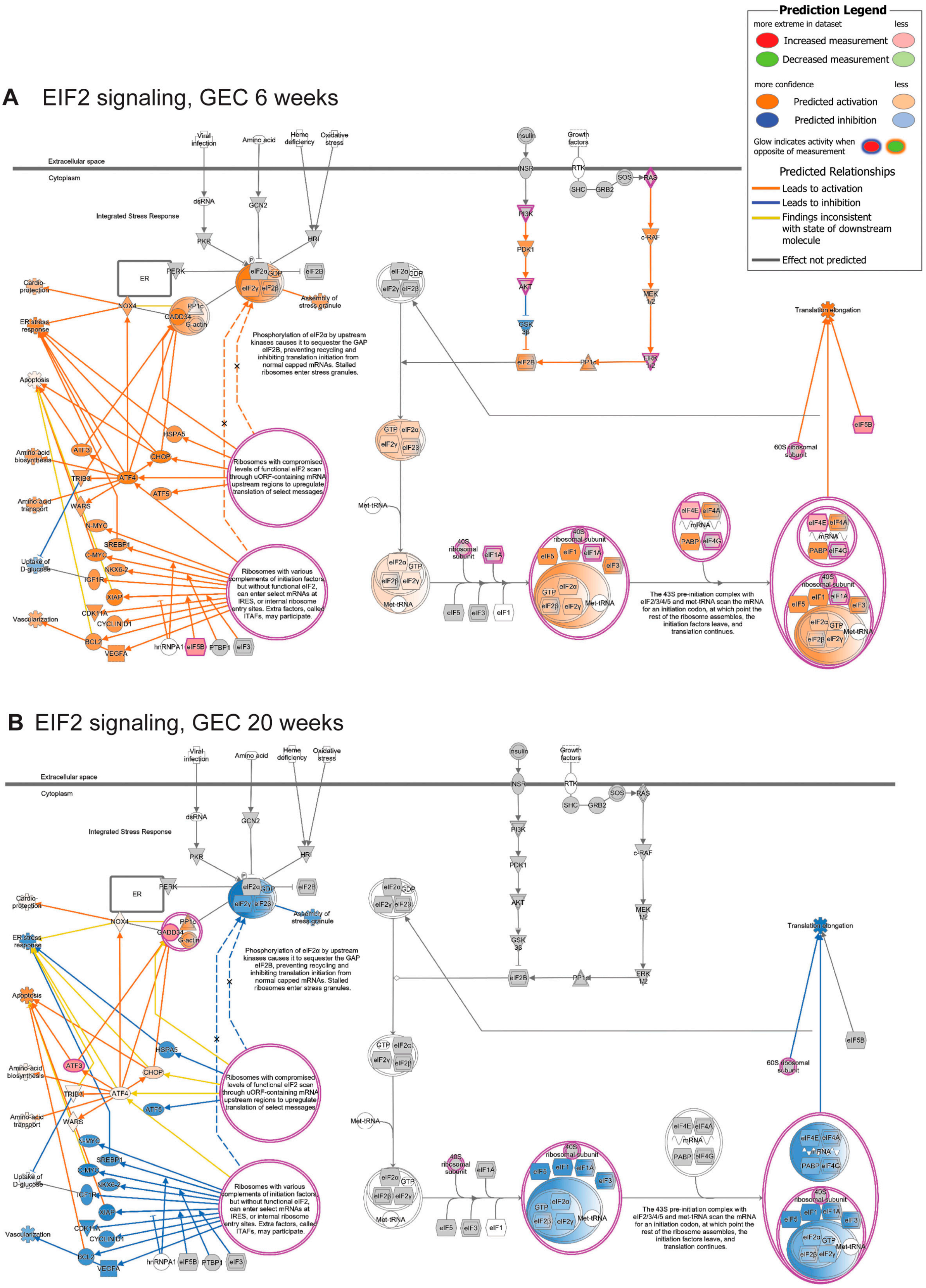

Figure S8. EIF2 signaling in BTBR*ob/ob* GEC at 6 and 20 weeks. Schematic diagram of EIF2 signaling in GEC at 6 weeks (A) and 20 weeks (B).

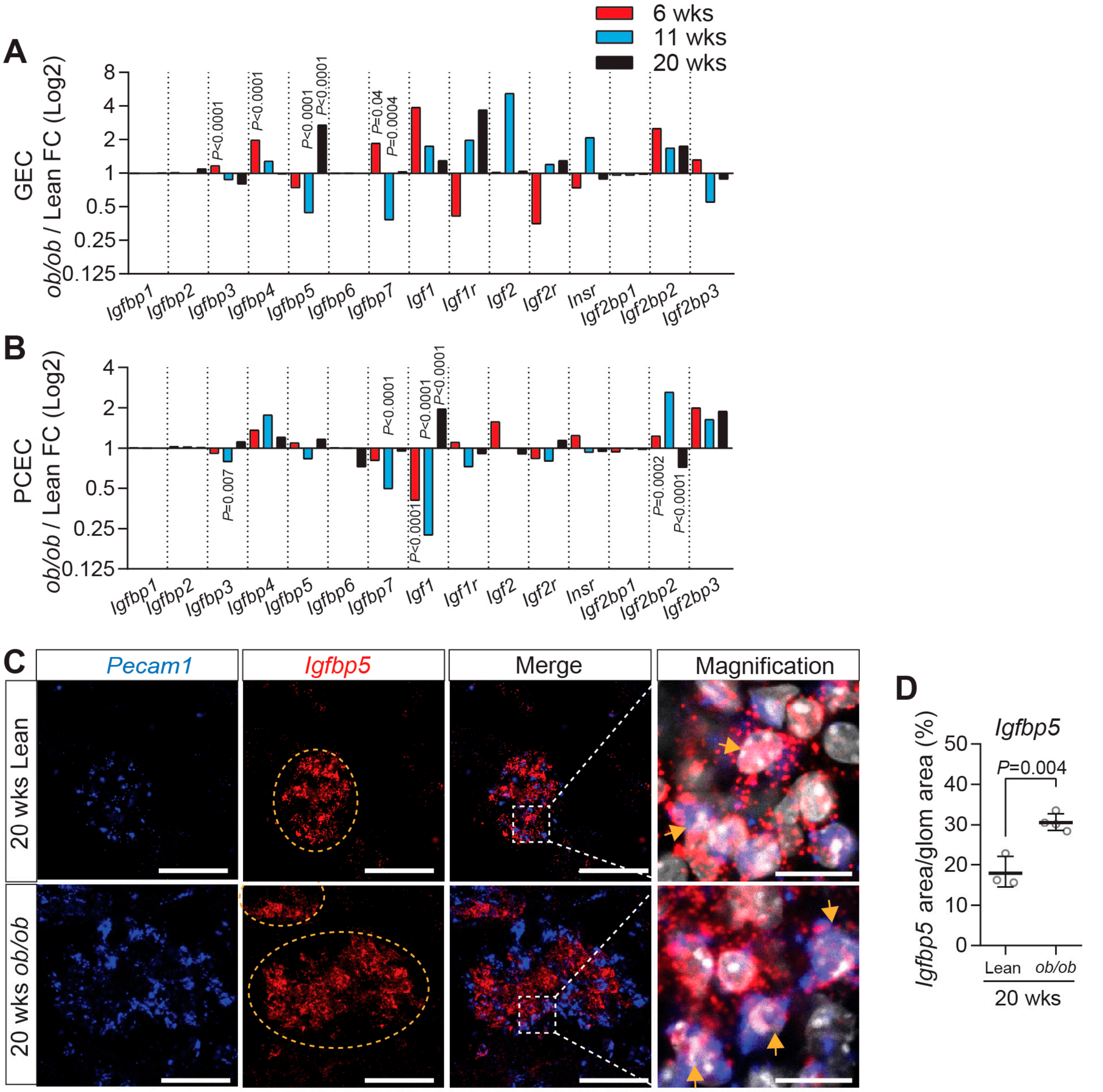

Figure S9. The IGF system in diabetic kidney disease in BTBR*ob/ob* mice. (A, B) Fold change expression of genes in the IGF system comparing non-diabetic (Lean) and diabetic (*ob/ob*) mice in GEC and PCEC, respectively. (C) RNA-ISH for glomerular expression of *Igfbp5* and *Pecam1* in 20-week-old non-diabetic (Lean) and diabetic (*ob/ob*) mice, glomeruli marked with orange circle. Orange arrows indicate *Pecam*+/*Igfbp5*+ cells. Scale bar = 50 µm, magnification scale bar = 10 µm. (D) Quantification of *Igfbp5* expression per glomerular area in non-diabetic (Lean) and diabetic mice (*ob/ob*) at 20 weeks. Data expressed as mean ± SD.

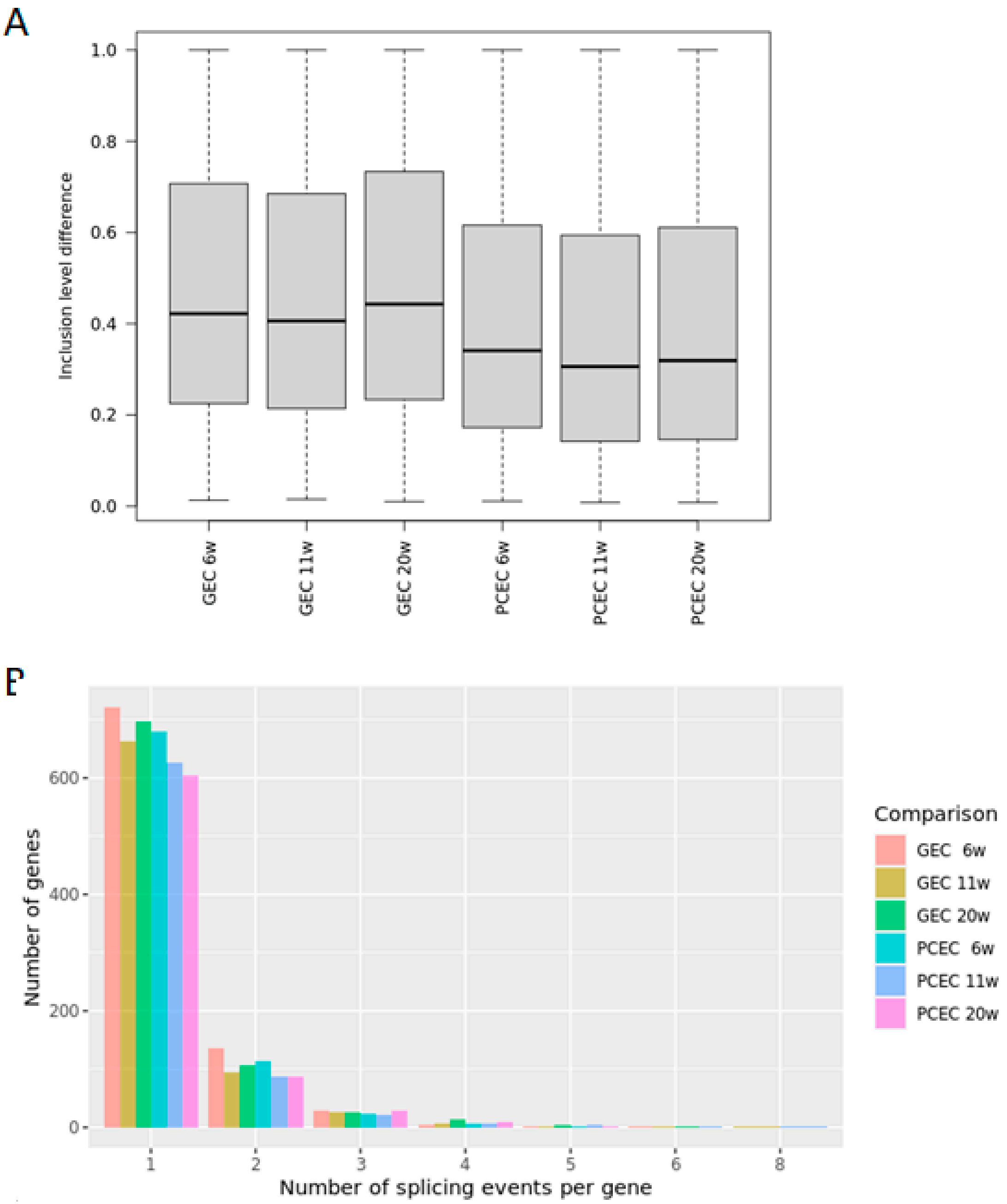

Figure S10. Characterization of DSEs in BTBR*ob/ob* mice. (A) Absolute inclusion level difference of DSEs (FDR<0.05) between BTBRLean and BTBR*ob/ob* GEC and PCEC at different timepoints. (B) Number of significant DSEs (FDR<0.05 and absolute inclusion level difference > 0.4) per gene.

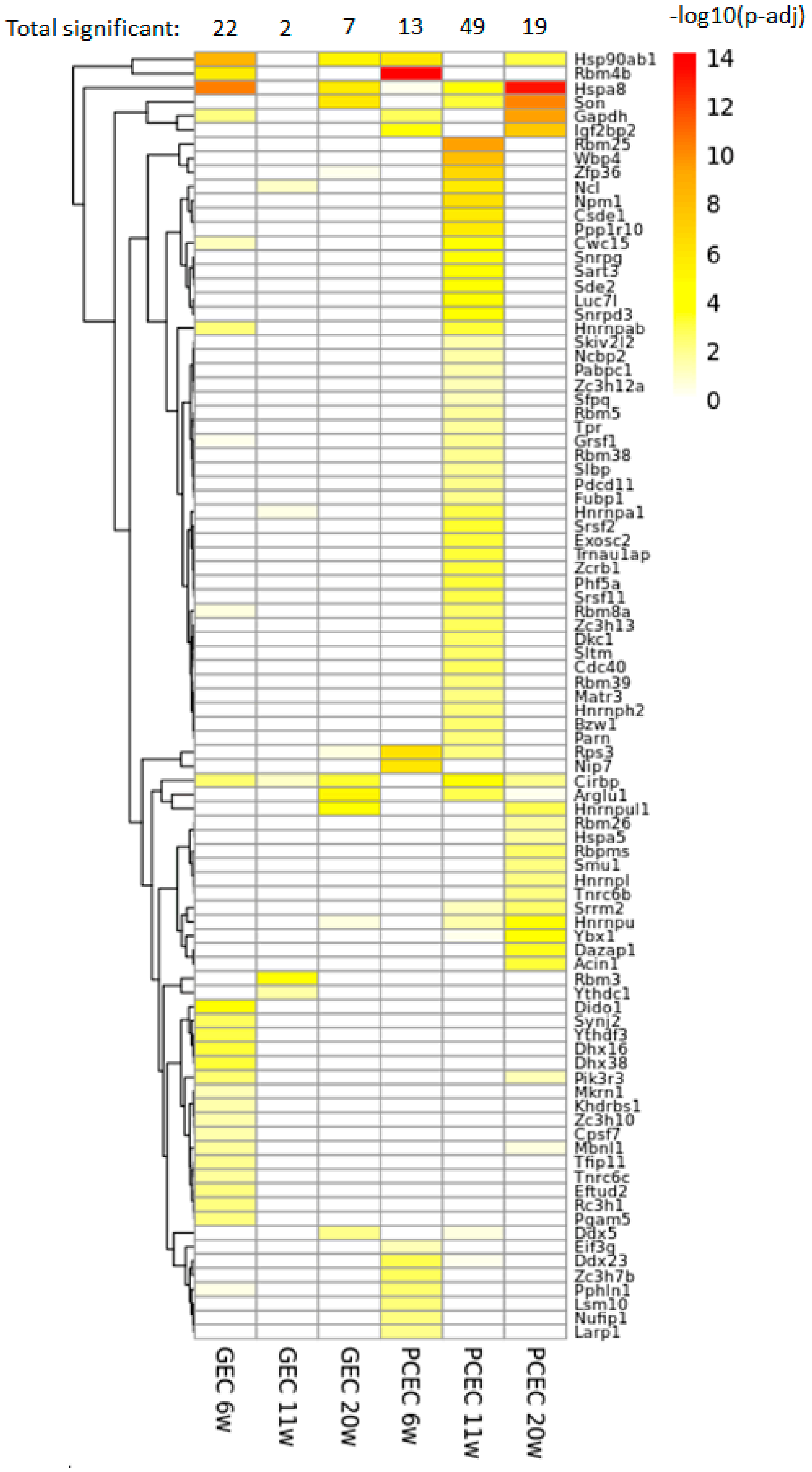

Figure S11. Overlapping BTBR*ob/ob* DEGs with a compiled list of mouse RBPs from 3 databases: rMAPS (http://rmaps.cecsresearch.org), RBPDB (http://rbpdb.ccbr.utoronto.ca), and Splicesome (http://spliceosomedb.ucsc.edu).

SUPPLEMENTAL TABLES

Table S1. Number of sequenced cells per cluster and timepoint

	6 wks		11 wks		20 wks	
Population	Lean	*ob/ob*	Lean	*ob/ob*	Lean	*ob/ob*
GEC	35	45	197	90	60	56
$GEC^{IEG\text{-}high}$	154	101	121	32	48	101
PCEC	210	130	555	526	276	298
$PCEC^{IEG\text{-}high}$	242	139	27	7	18	120
AA	57	32	49	17	17	43
EA	68	57	96	28	47	48
DVR	85	41	108	41	22	62
AVR	182	48	143	74	63	71
VEC	9	10	26	29	23	35
LVEC	3	62	5	2	2	0
Tip	7	4	27	6	3	21
Cycling	1	2	4	3	0	32
Unknown	5	4	17	12	9	14

GEC glomerular endothelial cells; *IEG* immediate early gene; *PCEC* peritubular capillary cells; *AA* arterioles/afferent arteriole; *EA* efferent arteriole; *DVR* descending vasa recta; *AVR* ascending vasa recta; *VEC* venous endothelial cells; *LVEC* lymphatic vascular endothelial cells; *Tip* cells

Table S2. Differentially expressed genes comparing Lean and *ob/ob* mice

	6 wks			11 wks			20 wks		
**Population**	**down**	**up**	**total**	**down**	**up**	**total**	**down**	**up**	**total**
GEC	121	618	739	62	39	101	151	34	185
PCEC	205	277	482	786	106	892	351	133	484
AA	0	2	2	0	0	0	0	2	2
EA	12	28	40	2	0	2	34	5	39
DVR	2	9	11	0	5	5	1	2	3
AVR	16	33	49	6	5	11	43	2	45
VEC	0	0	0	0	0	0	3	2	5

GEC glomerular endothelial cells; *PCEC* peritubular capillary cells; *AA* arterioles/afferent arteriole; *EA* efferent arteriole; *DVR* descending vasa recta; *AVR* ascending vasa recta; *VEC* venous endothelial cells

Table 3S. RNAscope probes and reagents

**Probe (mouse gene)**	**Type**	**Lot**	**Reference**
Mm-Calca	C1	18012B	420361
Mm-Gata5	C1	18205A	549061
Mm-Slc14a1	C1		448001
Mm-Cldn5	C3	19297B	491611-C3
Mm-Aqp1	C2	19179I	504741-C2
Mm-Igf1	C2		443901-C2
Mm-Sele	C1	19042A	438621
Mm-Car8	C2	19179I	514171-C2
Mm-Gper1	C3	19179I	475251-C3
Mm-Ace	C1	18218A	442731
Mm-Il33	C1		400591
Mm-Fos	C2	19176C	316921-C2
Mm-Junb	C1	18278B	556651
Mm-Atf3	C3	19179I	426891-C3
Mm-Pecam1	C3	18355C	316721-C3
Mm-Plvap	C1	19323A	440221
Mm-Plvap	C3	20072C	440221-C3
Mm-Acta2	C2	19316C	319531-C2
Mm-Acta2	C1	20281B	319531
Mm-Igfbp5	C1		425731
**Reagents**	**Type**	**Lot**	**Reference**
Protease III		2007018, 2002759	322340
3-plex Positive Control Probe			320881
3-plex Negative Control Probe			320871
Multiplex Fluorescent Reagent Kit v2		2007959, 2010572	323100
Multiplex Fluorescent Reagent		2007507	320851
Wash Buffer			310091
Opal520 Reagent		200106003	FP1487001KT
Opal570 Reagent		200106008	FP1488001KT
Opal690 Reagent		200106010	FP1497001KT

Table S4. Antibodies

Name (protein/antigen)	Company	Reference	Host	Lot	Dilution
Endomucin	Abcam	AB106100	rat		1:200
Plvap	BD Bioscience	550563	rat	9324203	1:200
Podocalyxin	R&D Systems	AF1556	goat		1:200
Pecam1-FITC	BD Bioscience	AF553370	rat	56813	1:200
Pecam1	Abcam	Ab28364	rabbit	GR3247742-11	1:200
Desmin	Abcam	Ab15200	rabbit		1:200
Collagen IV	BioRad	134001	goat		1:200
anti goat IgG-Alexa633	Thermo Fisher Scientific	A21082	donkey	1889311	1:200
anti rabbit IgG-Alexa680	Thermo Fisher Scientific	A10043	donkey	1917929	1:200
Anti rat IgG-Alexa633	Thermo Fisher Scientific	A21094		2002975	1:200
Hoechst33342	Thermo Fisher Scientific	H3570		1664690	1:1000
