# Supplementary material for: Renal Endothelial Single-Cell Transcriptomics Reveals Spatiotemporal Regulation and Divergent Roles of Differential Gene Transcription and Alternative Splicing in Murine Diabetic Nephropathy
Source: Int J Mol Sci. 2024 Apr 13;25(8):4320. doi: 10.3390/ijms25084320 (PMC11050020; doi:10.3390/ijms25084320)

Vascular segments – Canonical pathways

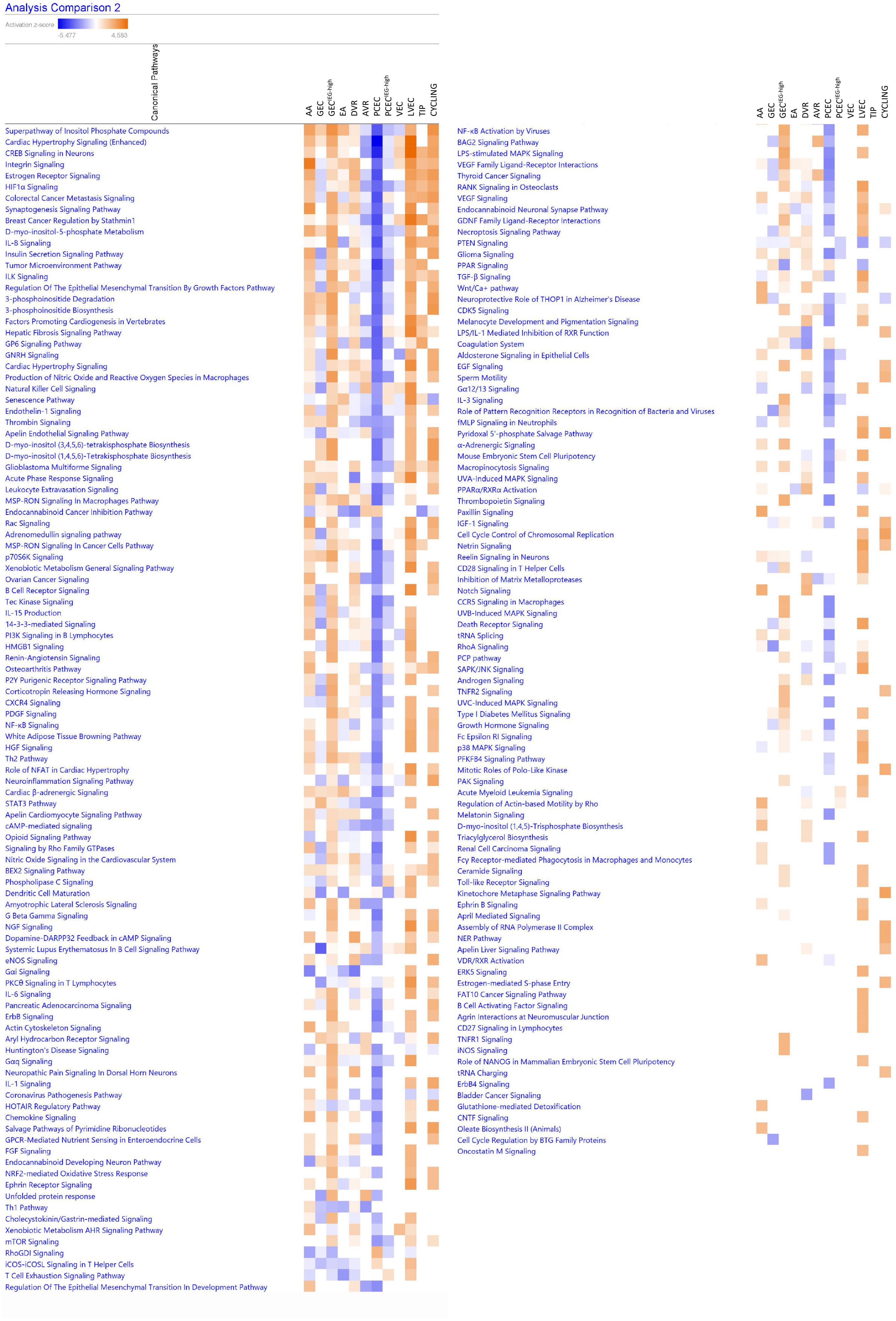

PCEC Canonical pathways

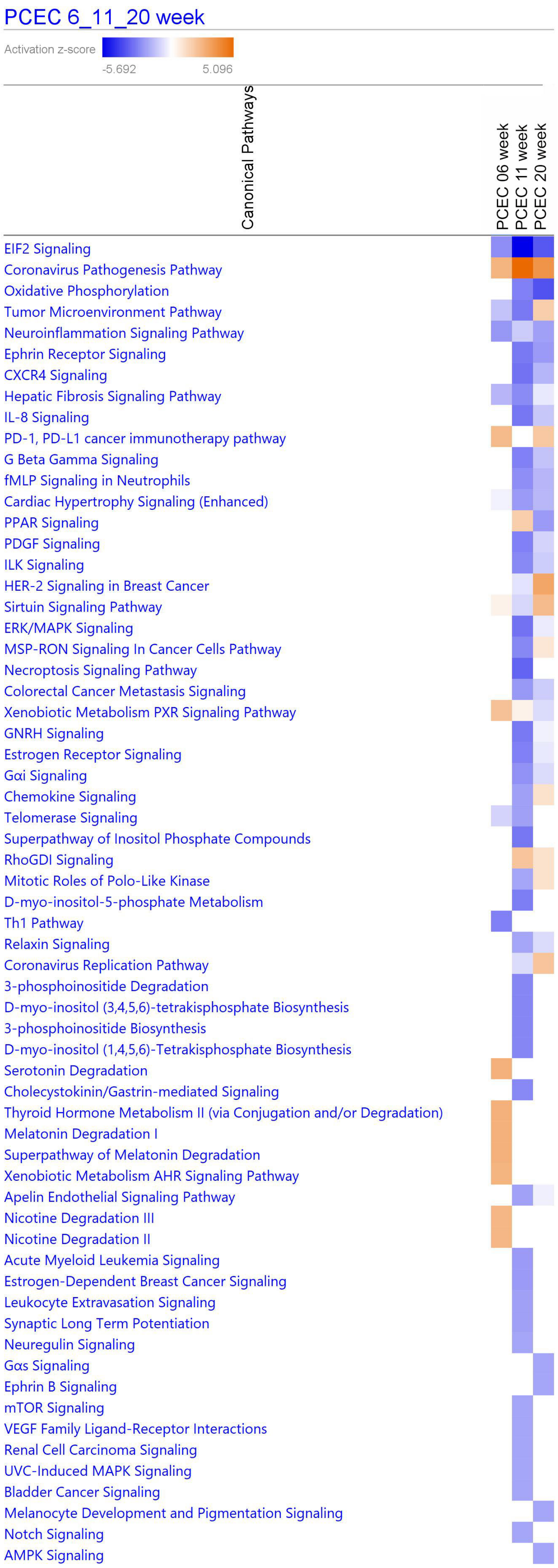

PCEC Downstream effects

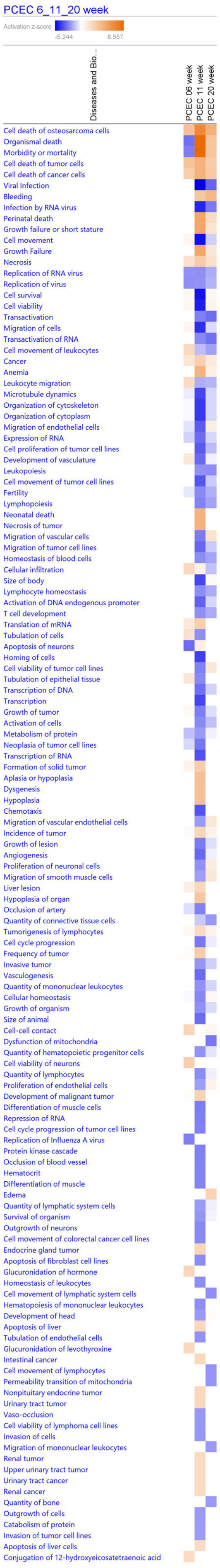

PCEC Upstream regulators – growth factors

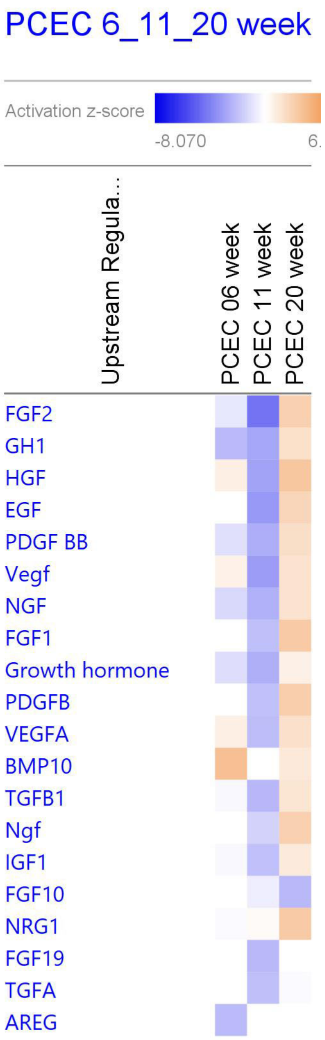

PCEC Upstream regulators – transcription

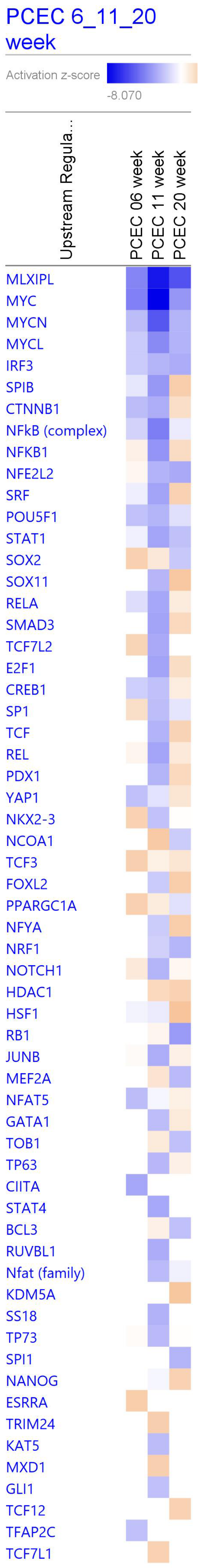

GEC Canonical pathways

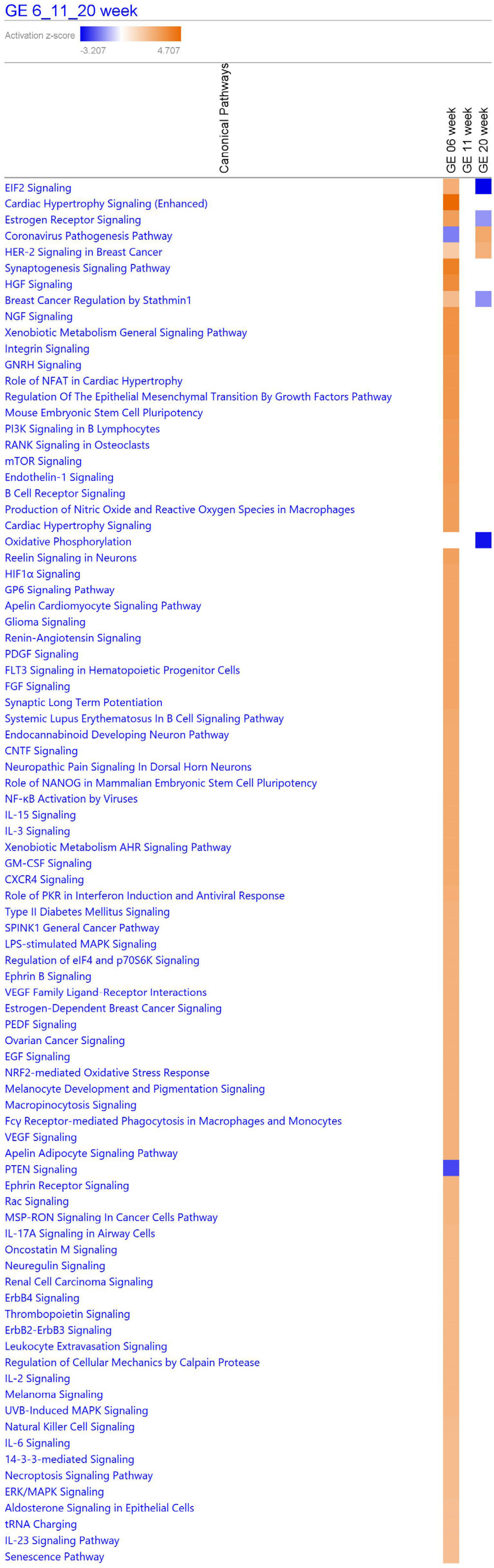

GEC Downstream effects

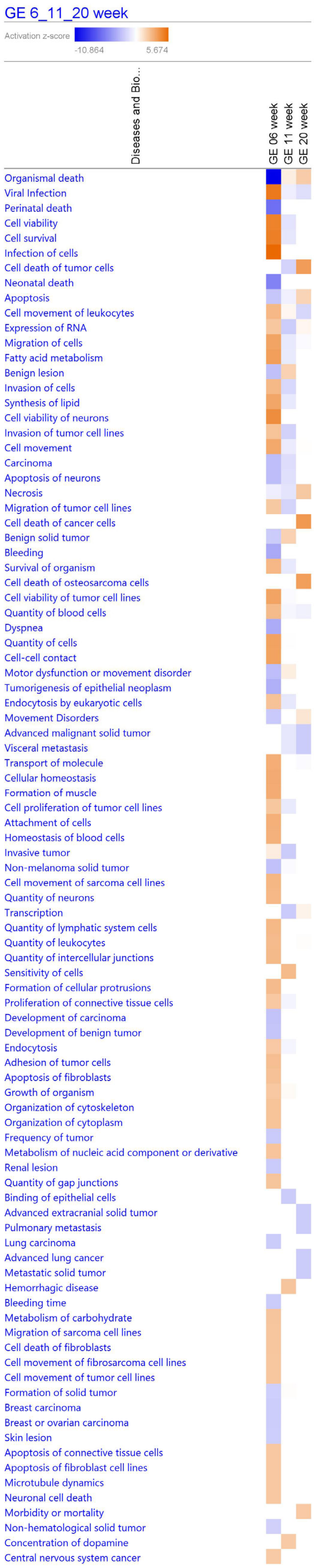

GEC Upstream regulators – growth factors

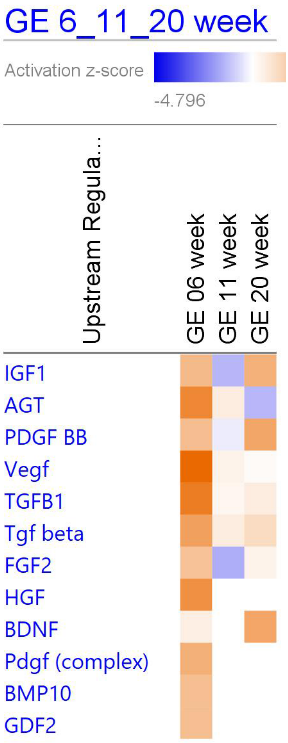

GEC Upstream regulator - transcription

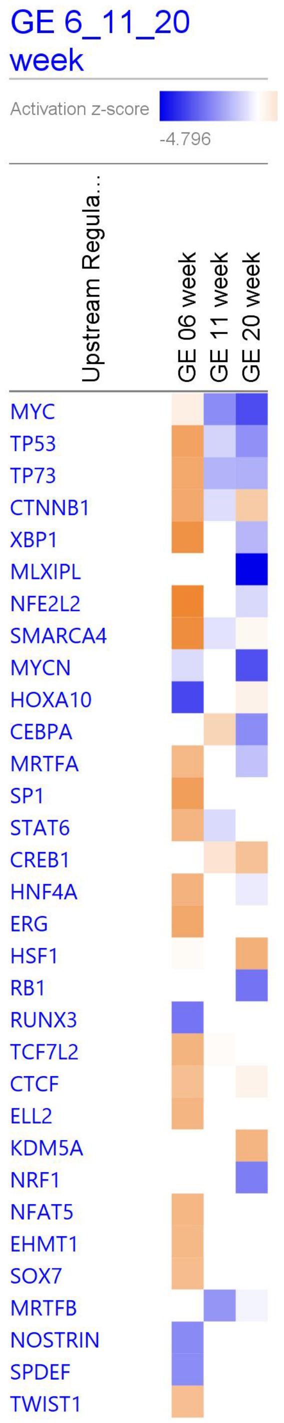

Supplement: Supplementary file 1 [file ijms-25-04320-s001.zip › Supplemental File S2_IPA analysis all data.pdf]
